# 2242. Leveraging the McGeer criteria to Determine the Frequency of Potentially Inappropriate Antibiotic Prescribing for Urinary and Respiratory Tract Infections Prior to and During the COVID-19 pandemic at a Skilled Nursing Facility

**DOI:** 10.1093/ofid/ofad500.1864

**Published:** 2023-11-27

**Authors:** Paulina M Colombo, Ferris A Ramadan, Dilsharan Kaur, Katherine Ellingson

**Affiliations:** University of Arizona Mel and Enid Zuckerman College of Public Health, TUCSON, Arizona; University of Arizona Mel and Enid Zuckerman College of Public Health, TUCSON, Arizona; Univeristy of Arizona Health Sciences, Tucson, Arizona; University of Arizona, Tucson, Arizona

## Abstract

**Background:**

Considerable reductions in antimicrobial stewardship efforts have been attributed to the COVID-19 pandemic, especially among low-resourced healthcare facilities, such as long-term care settings. Our study objectives were to (1) assess the appropriateness of antibiotic prescriptions for urinary (UTI) and respiratory tract infections (RTI) using standardized infection definitions, known as the McGeer criteria, and (2) to determine whether the prescribing practices differed prior to and during the COVID-19 pandemic.

**Methods:**

We employed a HIPPA-compliant REDCap data collection tool to abstract Electronic Medical Record data from an Arizona-based, Skilled Nursing Facility between March 2019 and January 2022. Clinical and microbiologic infection characteristics were abstracted to determine whether indications for antibiotic prescriptions met the McGeer criteria for UTI and RTI. The frequency and proportion of antibiotic prescriptions for suspected infections that did not meet McGeer criteria were compared for the time period prior to and during the COVID-19 pandemic.

**Results:**

388 total antibiotic events prescribed for UTI and RTI infections between March 2019 – January 2022 were analyzed (Figure 1). 61% (n=14) and 78% (n=154) of UTI prescriptions did not meet McGeer criteria prior to and during the COVID-19 pandemic, respectively. 70% (n=14) of RTI antibiotic prescriptions prior to the pandemic and 60% (n=89) of antibiotic events during the pandemic were not in accordance with McGeer criteria (Table 1) (Figure 2).

Flow diagram to demonstrate Electronic Medical Record abstraction analysis inclusion criteria

**Figure:**
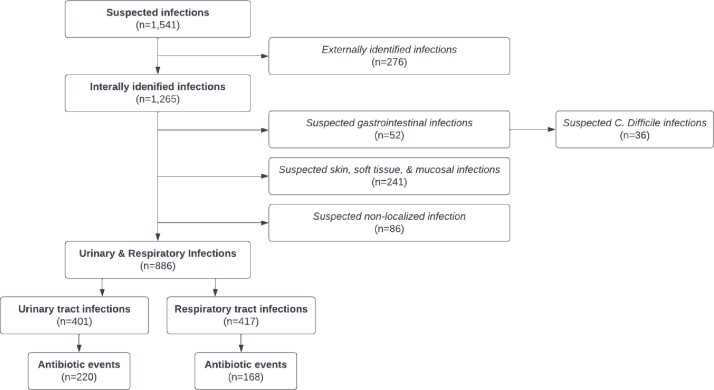

Note: a suspected infection was defined as a laboratory order (e.g., urine analysis, chest x-ray), or an antibiotic prescription

Frequency and percent of UTI and RTI antibiotic prescriptions that did not meet McGeer criteria, prior to and during the COVID-19 pandemic

**Figure:**
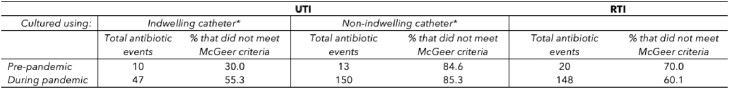

Note: suspected infections from March 01, 2019 - January 25, 2020 are considered “pre-pandemic,” and January 26, 2020 – January 23, 2022 are considered “during pandemic” *The McGeer criteria differentiate infection definitions based on the presence or absence of an indwelling catheter

Antibiotic prescription accordance with McGeer criteria from March 2019 – January 2022

**Figure:**
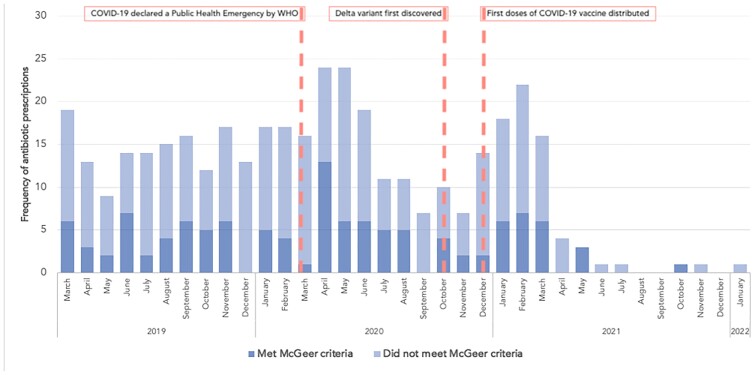

**Conclusion:**

Despite consorted efforts to improve antimicrobial stewardship infrastructure, the majority of antibiotic prescribing for UTIs and RTIs prior to the pandemic did not meet McGeer criteria. This trend also persisted during the pandemic, indicating a need for increased stewardship practices that can operate concurrently with future public health threats.

**Disclosures:**

**All Authors**: No reported disclosures

